# Interplay Between Plasma Glycine and Branched-Chain Amino Acids Contributes to the Development of Hypertension and Coronary Heart Disease

**DOI:** 10.1161/HYPERTENSIONAHA.123.22649

**Published:** 2024-04-08

**Authors:** Mateusz Dziedzic, Ewelina Józefczuk, Tomasz J. Guzik, Mateusz Siedlinski

**Affiliations:** 1 Department of Internal Medicine (M.D., E.J., T.J.G., M.S.), Faculty of Medicine, Jagiellonian University Medical College, Cracow, Poland.; 2 Center for Medical Genomics OMICRON (T.J.G., M.S.), Faculty of Medicine, Jagiellonian University Medical College, Cracow, Poland.; 3 Centre for Cardiovascular Science, Queen’s Medical Research Institute, University of Edinburgh, United Kingdom (T.J.G., M.S.).

**Keywords:** amino acids, branched-chain, glycine, hypertension, Mendelian randomization analysis, UK Biobank

## Abstract

**BACKGROUND:**

Higher levels of plasma glycine are linked to a reduced risk, while increased levels of total branched-chain amino acids (tBCAAs) are associated with a higher risk of essential hypertension and coronary heart disease (CHD). As these metabolic components are interconnected, analyzing the tBCAAs/glycine ratio may help to understand their interplay in the pathogenesis of cardiovascular disease.

**METHODS:**

The Cox regression approach was combined with the development of novel genetic tools for assessments of associations between plasma metabolomic data (glycine, tBCAAs, and tBCAAs/glycine ratio) from the UK Biobank and the development of hypertension and CHD. Genome-wide association study was performed on 186 523 White UK Biobank participants to identify new independent genetic instruments for the 2-sample Mendelian randomization analyses. *P*-gain statistic >10 identified instruments associated with tBCAAs/glycine ratio significantly stronger compared with individual amino acids. Outcomes of genome-wide association study on hypertension and CHD were derived from the UK Biobank (nonoverlapping sample), FinnGen, and CARDIoGRAMplusC4D.

**RESULTS:**

The tBCAAs/glycine ratio was prospectively associated with a higher risk of developing hypertension and CHD (hazard ratio quintile Q5 versus Q1, 1.196 [95% CI, 1.109–1.289] and 1.226 [95% CI, 1.160–1.296], respectively). Mendelian randomization analysis demonstrated that tBCAAs/glycine ratio (*P*-gain >10) was a risk factor for hypertension (meta-analyzed inverse-variance weighted causal estimate 0.45 log odds ratio/SD (95% CI, 0.26–0.64) and CHD (0.48 [95% CI, 0.29–0.67]) with an absolute effect significantly larger compared with the effect of glycine (−0.06 [95% CI, −0.1 to −0.03] and −0.08 [95% CI, −0.11 to −0.05], respectively) or tBCAAs (0.22 [95% CI, 0.09–0.34] and 0.12 [95% CI, 0.01–0.24], respectively).

**CONCLUSIONS:**

The total BCAAs/glycine ratio is a key element of the metabolic signature contributing to hypertension and CHD, which may reflect biological pathways shared by glycine and tBCAAs.

**NOVELTY AND RELEVANCE**

**What Is New?**

This study identified novel genetic variants associated with the plasma total branched-chain amino acids/glycine ratio.

**What Is Relevant?**

Mendelian randomization analysis found associations between genetically proxied plasma total branched-chain amino acids/glycine ratio and elevated risk for essential hypertension and coronary heart disease development beyond individual associations of each amino acid type.

**Clinical/Pathophysiological Implications?**

Identified metabolic signature of concurrently increased plasma levels of total branched-chain amino acids and reduced glycine may represent a valuable plasma clinical biomarker for identifying individuals at risk of hypertension and coronary heart disease, as observed in the prospective UK Biobank study.

Essential hypertension constitutes a major risk factor for a range of cerebrovascular and cardiovascular diseases and is partially determined by heritable traits.^1,2^ These include factors such as body mass index and dietary habits, as well as numerous molecular biomarkers.^2^

While metabolic and inflammatory signatures of hypertension and cardiovascular diseases exist^3^ and may accompany changes in clinical parameters of renal function,^4^ lipid balance,^4,5^ and immune profile,^3,6^ establishing their causal relationships with cardiovascular outcomes requires studies beyond the scope of observational research.^2^

Recent studies have identified particular amino acids as promising biomarkers of hypertension.^7^ Total branched-chain amino acids (tBCAAs), glycine, glutamine, histidine, and alanine, demonstrated associations with altered blood pressure (BP) and cardiometabolic diseases.^8,9^ This suggests that amino acids might modulate biochemical pathways of BP regulation.^8–12^ Mendelian randomization (MR) analysis, which utilizes genetic variations as instrumental variables (IVs), may deliver additional evidence on the cause-and-effect relationship between specific exposures and clinical outcomes in the field of cardiovascular disease research.^13–15^ Using MR analysis and phase 1 nuclear magnetic resonance metabolomics data from the UK Biobank, as well as data from the metabolomic study of Kettunen et al,^16^ Lin et al^8^ demonstrated that the level of glycine and BCAAs, being a pool of highly correlated valine, leucine, and isoleucine, may potentially causally associate with BP level and hypertension development, yet with the opposite direction of the effect. This is in line with other prospective and MR studies demonstrating potentially harmful effects of higher plasma BCAA levels, as well as lower plasma glycine levels on the development of hypertension, myocardial infarction, and coronary heart disease (CHD).^17–21^ Proinflammatory and pro-oxidant effects of tBCAAs, as opposed to the potentially counteracting effects of glycine, might provide an explanation for these associations.^22–24^

Given the fact that plasma metabolites, including amino acids, form a complex network of mutual relationships and participate in numerous biochemical cycles,^25^ analyzing their ratios may provide novel information on the genetic determinants of particular metabolites and their shared biology, together with links to the disease.^26^ Therefore, the current study, for the first time, aimed to identify novel genetic determinants of the tBCAAs/glycine ratio and to test whether this parameter is linked to the level of BP, the development of hypertension, and CHD beyond the individual association of each amino acid type.

## METHODS

### Data Availability

The UK Biobank data are available on application to the UK Biobank for data access (http://www.ukbiobank.ac.uk/). Genome-wide association study (GWAS) summary statistics from the FinnGen, CARDIoGRAMplusC4D, and UK Biobank+ International Consortium for Blood Pressure (ICBP) consortia are available at the MRC IEU OpenGWAS project.^27^ Other data that support the findings of this study are available from the corresponding author upon request.

### UK Biobank Study

Over 500 000 participants (aged 40–69 years) were recruited from 22 assessment centers throughout the United Kingdom between 2006 and 2010, encompassing a range of diverse environments to ensure a mix of socioeconomic backgrounds, ethnicities, and a balance between urban and rural settings.^28^ The study collected comprehensive information on the physical and genetic characteristics, including data obtained through surveys, physical assessments (see Supplemental Material for details on BP measurement procedures in the UK Biobank study), biological sample testing, genome-wide genotyping, and long-term monitoring of health-related results.^28^ The UK Biobank received ethical approval from the North-West Multi-Center Research Ethics Committee (11/NW/03820). All participants gave written informed consent before enrollment in the study, which was conducted according to the principles of the Declaration of Helsinki.

We have used UK Biobank phase 1 and 2 data of nuclear magnetic resonance biomarker data, quantified in ethylenediaminetetraacetic acid (EDTA) plasma and generated by Nightingale Health.^29^ Technical variation in the plasma level of glycine and tBCAAs was removed using the ukbnmr (version 2.0.1) R package.^30^ Plasma levels of tBCAAs were significantly correlated with levels of valine, leucine, and isoleucine (Spearman’s ρ >0.9) and glycine (Spearman’s ρ = −0.147) in the UK Biobank. We have performed prospective analyses linking baseline (2006–2010) plasma levels of tBCAAs, glycine, and their ratio with the development of hypertension (*International Classification of Diseases Tenth Revision* [*ICD10*] code I10, UK Biobank field ID 131286) and CHD (*ICD10* codes I20–I25, UK Biobank field IDs: 131296–131307). This analysis was performed using Kaplan-Meier survival analysis and Cox regression model adjusted for baseline age, sex, body mass index, smoking status, and alcohol intake frequency in participants with no prior history of the disease. Additionally, the analysis of hypertension was restricted to normotensive participants (average automated reading of systolic BP [SBP] <140 mm Hg and diastolic BP [DBP] <90 mm Hg, no BP-lowering medications reported) at baseline, and sex-specific analyses have been performed. Sensitivity analyses included additional covariates, that is, the Townsend deprivation index, the weekly frequency of moderate physical activity (hypertension and CHD analyses), and the use of insulin, BP-, and cholesterol-lowering medications (CHD analysis) at baseline. The censoring time was set to October 31, 2022, the date of death or the date of the hypertension/CHD record, whichever occurred first. Competing-risks regression analysis took into account death as a competing event and was additionally performed to validate Cox regression results.

### GWAS in the UK Biobank

We excluded subjects with a missing genotype rate >10% or an amino acid level 10× below the median, similarly to a previously published study.^25^ To approximate normal distribution, all 3 amino acid outcome variables were log10 transformed; this has additionally made ratio analyses independent of the amino acid used as numerator/denominator. We performed GWAS on amino acid levels on 186 523 unrelated, White UK Biobank participants using GLM procedure implemented in PLINK2 software and adjusting for age, sex, the top 10 principal components of genetic ancestry, and genotyping batch (UK Biobank versus UK BiLEVE). See Supplemental Material for details regarding GWAS on SBP/DBP level, hypertension, and CHD in the UK Biobank subsample with no subjects overlapping with amino acid GWAS.

### Two-Sample MR Analysis Testing Potentially Causal Effects of Plasma Level of Glycine, tBCAAs, and Their Ratio on Cardiovascular Outcomes

We excluded single-nucleotide polymorphism (SNPs) based on minor allele frequency (MAF) <0.01, genotyping call rate <90%, and Hardy-Weinberg equilibrium *P* value threshold <10^−8^. Independent (r^2^<0.001) genetic instruments for amino acid exposure variables were obtained using clumping procedure against the European panel available in ieugwasr (version 1.0.5) R package and annotated using wANNOVAR.^31,32^ All instruments demonstrated association with exposure traits at *P*<5×10^−8^ with an F statistics >10. Furthermore, we excluded instruments, which did not retain association at *P*<5×10^−8^ in an analysis conditioned on the top SNP within ±1 Mb range (±4 Mb for the *CPS1* [carbamoyl-phosphate synthase 1] locus). None of the genetic instruments used were associated with the outcome being stronger compared with exposure based on the Steiger test at nominal *P*<0.01 and using an effective sample size^33^ for case-control studies using TwoSampleMR R package.^27,34,35^ Additional MR analysis used genetic instruments for the analyzed ratio with a *P*-gain value >10, that is, the *P* value of their association with the ratio was at least 10× lower than the lowest *P* value of their association with the level of either glycine or tBCAAs.^26^ MR analyses rely on 3 key assumptions: relevance (genetic variant, used as IV, associates with exposure, that is, amino acid level), independence (lack of unmeasured confounders of the association between IV and outcome), and exclusion restriction (IV affects the outcome only through the exposure of interest).^15^ Primary MR analyses were performed using the most powerful inverse-variance weighted (IVW) method. Sensitivity MR analyses allow relaxing certain assumptions, such as lack of genetic pleiotropy, and were performed using a weighted median approach, allowing up to 50% of the weights to be invalid IVs; the MR-Egger method, which can include SNPs with pleiotropic effects that are not proportional to the effects of these SNPs on exposure; and the MR-PRESSO (Mendelian Randomization Pleiotropy Residual Sum and Outlier) method, which identifies and excludes SNPs that most likely display pleiotropic effects.^15,36^ Estimates of the effect of SNPs on amino acid and ratio levels were transformed to SD units. The outcome of the SBP/DBP GWAS used for MR was derived from the study of Evangelou et al^37^ (757 601 subjects from the UK Biobank and ICBP) and the UK Biobank (see Supplemental Material for details). The latter GWAS did not adjust for body mass index and had no overlapping subjects in contrast to the more powerful GWAS of Evangelou et al. We have complemented MR analyses with assessing the effects of genetically proxied amino acids on hypertension in the FinnGen^38^ (r5, 42 857 cases and 162 837 controls) and UK Biobank (nonoverlapping sample) as well as coronary artery disease in the CARDIoGRAMplusC4D Consortium^39^ (60 801 cases and 123 504 controls), CHD in FinnGen (31 640 cases and 187 152 controls) and UK Biobank (nonoverlapping sample). Absolute values of causal MR estimates corresponding to different amino acid traits were compared using *Z* test and a critical *Z* score value of 1.96.^40^

### Software

GWAS analyses in the UK Biobank were performed with PLINK2.^41^ Observational analyses and meta-analyses of MR effect sizes were performed using SPSS (version 29.0) and Graphpad Prism (version 10). MR analyses were performed using TwoSampleMR^34,35^ (version 0.5.7), MR-PRESSO,^36^ and MendelianRandomization^42^ packages in R (version 4.3.1) with default settings. MRC IEU OpenGWAS project^27^ was used as a source of GWAS performed in the FinnGen, CARDIoGRAMplusC4D, and UK Biobank+ICBP consortia.

## RESULTS

### Prospective Links Between Investigated Amino Acids and Development of Hypertension and CHD

**Figure 1.**
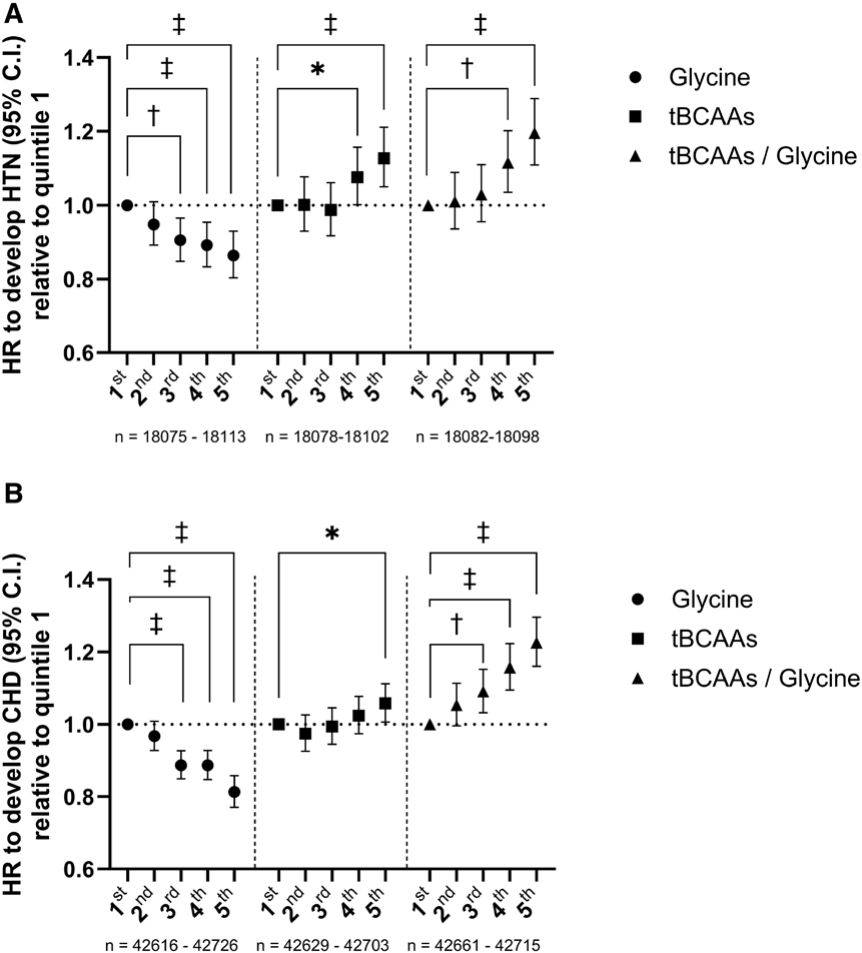
**Adjusted risk to develop hypertension (HTN) among normotensive subjects and coronary heart disease (CHD) according to amino acid quintiles in the UK Biobank study. A**, Analyses were performed in 90 446 normotensive participants (average automated systolic blood pressure and diastolic blood pressure readings below 140 and 90 mm Hg, respectively, no blood pressure–lowering treatment at baseline) using Cox regression adjusted for sex, age, body mass index, smoking status, and alcohol intake frequency. In total, 8703 participants developed *International Classification of Diseases Tenth Revision* (*ICD10*)-defined hypertension during follow-up. **B**, Analyses were performed in all UK Biobank participants with data on amino acid level available using Cox regression adjusted for sex, age, body mass index, smoking status, and alcohol intake frequency. In total, 18 293 participants developed *ICD10*-defined CHD (codes: I20–I25). HR indicates hazard ratio; and tBCAAs, total branched-chain amino acids. *P<0.05; ^†^*P*<0.01; ^‡^*P*<0.001.

Analyses focused on 227 560 White participants with available data on plasma levels of glycine, tBCAAs, and tBCAAs/glycine ratio (Table). A Cox regression analysis of 90 446 subjects who were normotensive at baseline indicated that elevated levels of glycine significantly correlated with a decreased hazard of developing hypertension (Figure 1A). In contrast, higher levels of tBCAAs and the tBCAAs/glycine ratio were associated with a significantly increased hazard ratio of developing hypertension (Figure 1A). Similarly, increased plasma glycine level was associated with a reduced risk of CHD in the entire UK Biobank cohort with amino acid data available (Figure 1B). In contrast, an elevated tBCAAs/glycine ratio was correlated with an increased risk of CHD, while the detrimental association with tBCAA levels alone was significant for the fifth quintile only (Figure 1B). The above results were confirmed using competing-risks regression analysis, which took into account death as a competing event (Table S1), Kaplan-Meier analysis (Figure S1A through S1F), and after additional adjustment for physical activity, Townsend deprivation index, intake of medications, and SBP (Figure S2A through S2D). Interestingly, a significant association of tBCAAs with the risk of developing essential hypertension, but not CHD, was observed in subjects with the lowest level of plasma glycine exclusively (Figure S3A and S3B), which may indicate that a high tBCAAs/low glycine metabolic signature may be of particular importance for the development of hypertension. The effects of amino acid quintiles on the risk of developing hypertension were similar in both sexes, which was additionally confirmed by analyses of appropriate interaction terms (Figure S4A through S4I), while only the effect of tBCAAs on CHD significantly differed between sexes with a more detrimental impact observed in women (Figure S5A through S5I).

**Table.**
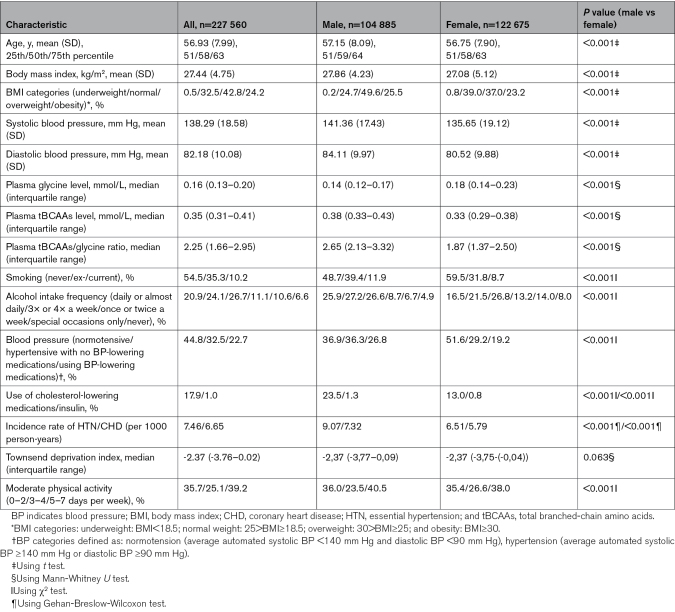
Baseline Characteristics of 227 560 UK Biobank Participants

### Genetic Evidence of an Association Between Plasma Glycine, tBCAAs, and Their Ratio With BP Levels and the Development of Hypertension

GWAS identified 57 conditionally independent SNPs associated with plasma glycine level at *P*<5×10^−8^ in 186 523 unrelated White UK Biobank subjects (Table S2). Similarly to previous studies, the top SNP associated with glycine level was located in the *CPS1* (missense rs1047891 SNP, *P*=10^−6588.16^), which explains the vast majority of the glycine level variation compared with other genetic variants^18^ identified. GWAS on tBCAAs identified 33 independent SNPs, while GWAS on tBCAAs/glycine ratio identified 61 independent SNPs, including 22 SNPs characterized by a *P*-gain value of >10 (Tables S3 and S4).

**Figure 2.**
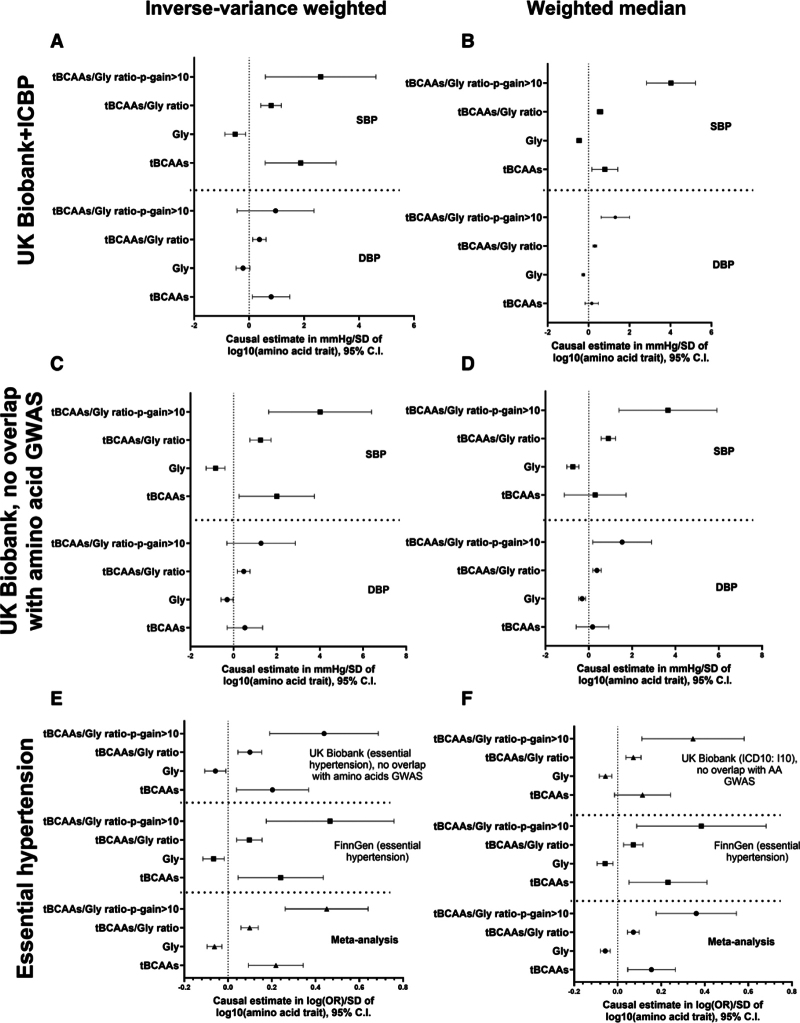
**Effects of genetically proxied amino acid traits on the level of blood pressure and development of hypertension.** Inverse-variance weighted (**A**, **C**, and **E**) and weighted median (**B**, **D**, and **F**) Mendelian randomization methods were used to calculate causal estimates corresponding to the effects of glycine, plasma total branched-chain amino acids (tBCAAs) and tBCAAs/glycine ratio on the level blood pressure and development of hypertension. Analyses used independent (r^2^<0.001) genetic instruments derived from genome-wide association study (GWAS) performed in 186 523 unrelated, White UK Biobank participant (Tables S2 through S5). A *P*-gain statistic >10 was used to identify genetic instruments specific for tBCAAs/glycine ratio. Outcome data were derived from meta-analysis of International Consortium for Blood Pressure and UK Biobank (GWAS on systolic blood pressure [SBP]/diastolic blood pressure [DBP]; **A** and **B**), the UK Biobank (SBP/DBP no overlapping subjects with amino acid GWAS; **C** and **D**), and the UK Biobank (no overlapping subjects with amino acid GWAS) and FinnGen (**E** and **F**). OR indicates odds ratio.

MR analyses used conditionally independent SNPs as IVs (Tables S2 through S5). Analyses of continuously defined SBP levels in the UK Biobank+ICBP consortium revealed a significant BP-increasing effect of genetically determined tBCAA levels and tBCAAs/glycine ratio and a potentially protective effect of glycine level using the IVW method (Figure 2A). The effects on DBP were largely consistent, yet less pronounced, compared with SBP analysis (Figure 2A). Results were confirmed using weighted median and MR-PRESSO methods (Figure 2B; Figure S6A). Except for the association between tBCAAs and SBP, similar results were obtained using a subsample of the UK Biobank, with no subjects overlapping with amino acid GWAS (Figure 2C and 2D; Figure S6C). Importantly, consistent results were obtained using a dichotomously defined hypertension both in the UK Biobank and FinnGen, where genetically proxied tBCAAs and tBCAAs/glycine ratio were risk factors for the disease while level of glycine protected against hypertension development (Figure 2E and 2F; Figure S6E and S6F). The MR-Egger method provided estimates of similar direction compared with the IVW method (Figure S6B and S6D) with no evidence of horizontal pleiotropy as assessed by the intercept analysis except for the tBCAAs/glycine ratio analyzed with all available instruments (Table S6). While the MR-Egger method is characterized by a relatively low power compared with other MR methods,^43^ we found a significant, potentially causal effect of the tBCAAs/glycine ratio, defined using genetic instruments with a *P*-gain >10, on hypertension in the UK Biobank subsample and meta-analyzed FinnGen and UK Biobank subsample data sets using this sensitivity analytical approach (Figure S6F).

Of note, while the absolute effect of tBCAAs was relatively larger compared with the absolute effect of glycine on BP and hypertension across various MR methods, absolute effect of tBCAAs/glycine ratio, when analyzed using genetic instruments with a *P*-gain value >10, was even larger when compared with the absolute effect of any other single amino acid trait analyzed. For example, a *Z* test for the difference in absolute effect sizes (ratio [*P*-gain >10] versus glycine and ratio [*P*-gain >10] versus tBCAAs) demonstrated a critical *Z* score >1.96 for analysis using continuously defined SBP and weighted median method in both the UK Biobank+ICBP consortium and the UK Biobank subsample with no subject overlap with amino acid GWAS. Similarly, the effect of tBCAAs/glycine ratio (*P*-gain >10) on hypertension in meta-analyzed FinnGen and UK Biobank was significantly larger than the independent effects of either tBCAAs or glycine using IVW, MR-Egger, and MR-PRESSO methods.

**Figure 3.**
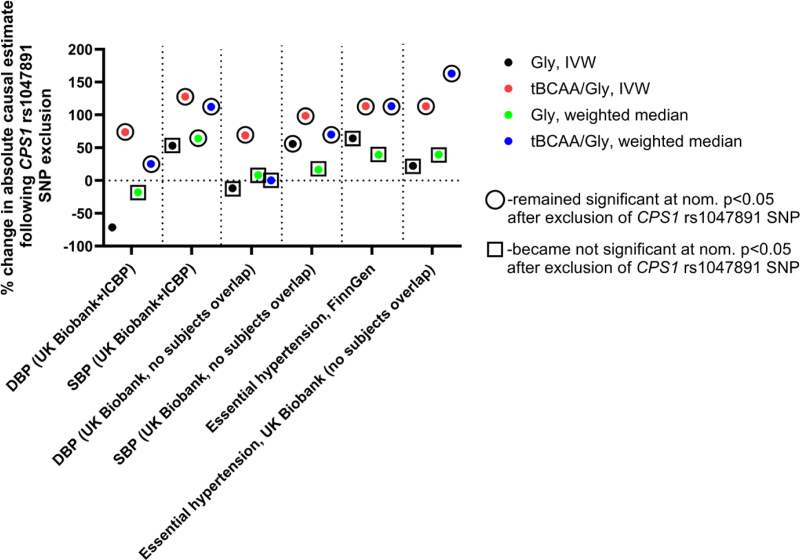
**Effect of rs1047891 *CPS1* (carbamoylphosphate synthase 1) single-nucleotide polymorphism (SNP) exclusion on causal estimates derived from Mendelian randomization analyses on blood pressure level and development of hypertension.** Inverse-variance weighted (IVW) and weighted median Mendelian randomization methods were used to calculate change in absolute causal estimates following exclusion of *CPS1* SNP from the instrument sets specific for glycine and total branched-chain amino acids (tBCAAs)/glycine ratio (Tables S2, S4, and S5). DBP indicates diastolic blood pressure; Gly, plasma glycine level; ICBP, International Consortium for Blood Pressure; and SBP, systolic blood pressure.

Absolute causal estimates of the effects of tBCAAs/glycine ratio, defined using all available instruments irrespective of *P*-gain value, were similar to those obtained for glycine, likely due to the presence of highly significant *CPS1* variants in instrument sets of both traits (Tables S2 and S4). Leave-one-out analyses IVW analyses demonstrated that tBCAAs and tBCAAs/glycine ratio (*P*-gain >10) analyses were not driven by any single variant (Table S7). Analyses of plasma glycine and tBCAAs/glycine ratio (all instruments) identified the *CPS1* rs1047891 SNP, which significantly affected causal estimates obtained (Figure 3; Table S7). For example, exclusion of the rs1047891 SNP generally yielded larger absolute causal estimates for glycine and especially tBCAAs/glycine ratio (Figure 3); however, it resulted in a significant loss of causal estimate of the effect of glycine level on hypertension (in both FinnGen and UK Biobank subsamples) or SBP level in the UK Biobank-ICBP meta-analysis (Figure 3; Table S7). Importantly, *CPS1* rs1047891 SNP was not identified as an instrument for tBCAAs/glycine ratio with a *P*-gain value >10. Exclusion of *CPS1* SNP had no influence on *Z* test results comparing absolute effect sizes of plasma glycine and tBCAAs/glycine ratio (*P*-gain >10) levels on SBP and hypertension mentioned above.

### MR Analyses on the Association Between Glycine, tBCAAs, and Their Ratio With CHD

**Figure 4.**
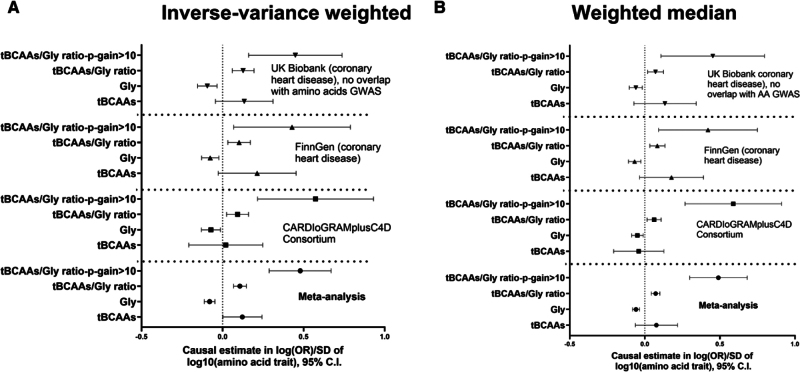
**Effects of genetically proxied amino acid traits on the development of coronary heart disease (CHD).** Inverse-variance weighted (**A**) and weighted median (**B**) Mendelian randomization methods were used to calculate causal estimates corresponding to the effects of glycine, total branched-chain amino acids (tBCAAs) and tBCAAs/glycine ratio on the development of CHD. Analyses used independent (r^2^<0.001) genetic instruments derived from genome-wide association study (GWAS) performed in 186 523 unrelated, White UK Biobank participant (Tables S2 through S5). A *P*-gain statistic >10 was used to identify genetic instruments specific for tBCAAs/glycine ratio. Outcome data were derived from UK Biobank (no overlapping subjects with amino acid GWAS) FinnGen and CARDIoGRAMplusC4D consortium. Gly indicates plasma glycine level; and OR, odds ratio.

**Figure 5.**
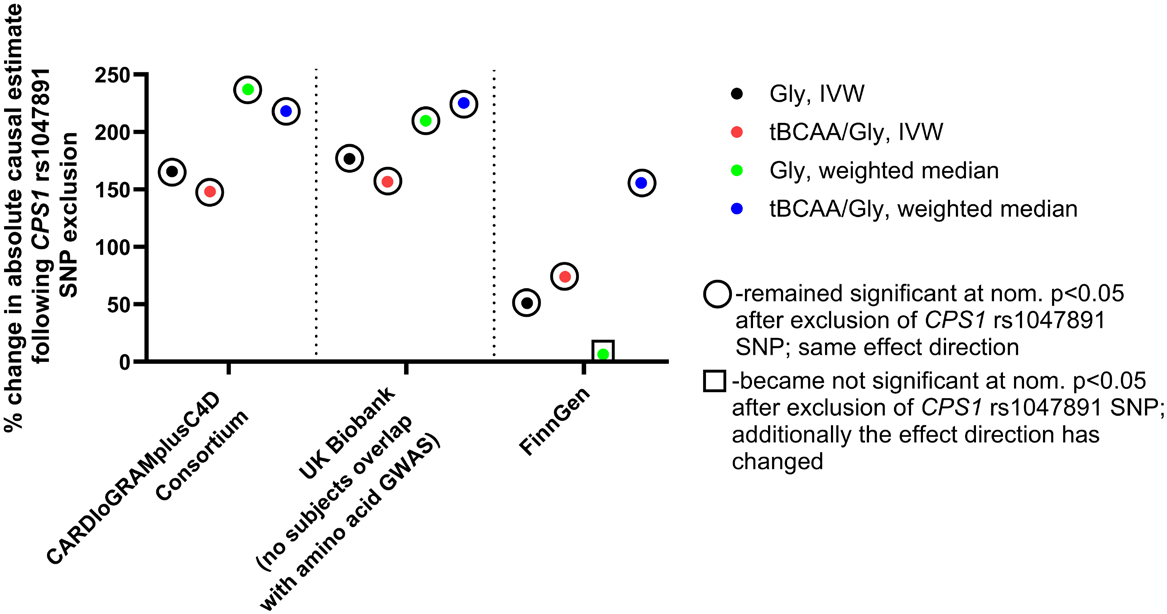
**Effect of rs1047891 *CPS1* (carbamoylphosphate synthase 1) single-nucleotide polymorphism (SNP) exclusion on causal estimates derived from Mendelian randomization analyses on the development of coronary heart disease.** Inverse-variance weighted (IVW) and weighted median Mendelian randomization methods were used to calculate change in absolute causal estimates following exclusion of *CPS1* SNP from the instrument sets specific for glycine and total branched-chain amino acids (tBCAAs)/glycine ratio (Tables S2, S4, and S5). Gly indicates plasma glycine level; and GWAS, genome-wide association study.

Our MR analyses on CHD encompassed 3 large and independent GWAS derived from the CARDIoGRAMplusC4D consortium focused on coronary artery disease, FinnGen and UK Biobank subsample with no subject overlap compared with amino acid GWAS, both defining CHD based on ICD10 coding (I20–I25). MR analyses demonstrated a consistent, protective effect of genetically defined glycine levels on CHD development across 3 cohorts using IVW and weighted median methods (Figure 4A and 4B). The level of tBCAAs was a risk factor for CHD, yet this effect did not reach statistical significance in either of the cohorts analyzed (Figure 4A and 4B). Importantly, the tBCAAs/glycine ratio, defined with a genetic IV with a *P*-gain >10, demonstrated a consistently larger absolute effect on CHD development compared with other amino acid traits (Figure 4A and 4B). *Z* test confirmed that this effect was significantly larger (critical *Z* score >1.96) in the CARDIoGRAMplusC4D cohort, as well as in the meta-analysis of all 3 cohorts using IVW, weighted median, and MR-PRESSO methods (Figure 4A and 4B; Figure S7A). MR-Egger analysis found a consistent direction of the effects for all amino acid traits analyzed compared with other MR methods, yet the effects identified were not significant (Figure S7B). The MR-Egger intercept test found evidence for horizontal pleiotropy predominantly in tests involving glycine and tBCAAs/glycine ratio (all instruments; Table S6). Exclusion of *CPS1* rs1047891 variant resulted in loss of significance for the MR-Egger intercept and had a significant impact on causal estimates obtained (Figure 5; Table S7). For example, in the CARDIoGRAMplusC4D and UK Biobank studies, absolute causal estimates increased more than 2-fold and became even more significant in MR analysis involving glycine and tBCAAs/glycine ratio (all instruments) levels (Figure 5; Table S7). However, the absolute effect of tBCAAs/glycine ratio (*P*-gain >10) on CHD was significantly larger compared with the effect of glycine, estimated without *CPS1* variant, in the CARDIoGRAMplusC4D and in meta-analyzed data set of the 3 cohorts (IVW, weighted median, and MR-PRESSO methods).

## DISCUSSION

Our study found that the plasma tBCAAs/glycine ratio was a metabolic signature constituting a risk factor for hypertension, CHD, and increased BP levels beyond individual associations of tBCAAs and glycine, which may reflect biological pathways shared by both amino acid types. This has been observed primarily in MR analyses using genetic instruments specific for the above ratio parameter and across various MR sensitivity methods, while prospective observational analyses confirmed the direction of effect provided by all amino acid traits analyzed.

The MR approach, which can mimic randomized controlled trials when all assumptions hold, allowed for the characterization of the potentially causal association between elevated plasma tBCAAs and reduced glycine levels with an increased risk of developing hypertension and CHD.^8,18^ The above associations were confirmed in the present study, which performed the GWAS using phase 2 metabolomic data from the UK Biobank to identify independent instruments for plasma levels of glycine and tBCAAs. Our results suggest that the absolute effect of tBCAAs/glycine ratio, defined with instruments with a *P*-gain >10, on hypertension or CHD is significantly stronger compared with the individual absolute effects of glycine and tBCAAs, and this observation holds after the exclusion of a *CPS1* instrument, which is characterized by sex-specific effects,^18^ and thus may additionally influence MR results. Various sensitivity MR methods confirmed the obtained results, especially with regard to hypertension, demonstrating a significant causal estimate of tBCAAs/glycine ratio (*P*-gain >10) on hypertension using relatively low-powered MR-Egger approach.

While glycine is synthetized endogenously mainly in the liver and kidneys from serine, choline, threonine, hydroxyproline, and glyoxylate, tBCAAs are essential amino acids that are delivered with the diet.^24,44^ Besides protein-building properties, these amino acids possess distinct signaling capabilities, including glycine action as a neurotransmitter or tBCAAs as a stimulus for mTOR (mammalian target of rapamycin) pathway activation.^24,44^ The network of the synthesis and metabolism of amino acids comprises intricate pathways that are interconnected^25^; thus, alterations in the metabolism of 1 amino acid may potentially lead to a cascade of associated events affecting other metabolite levels and, in consequence, their downstream signaling.

Studies strongly support a dynamic interaction between glycine and tBCAAs. For example, a growing body of evidence suggests that a concurrent low glycine/elevated tBCAAs profile may be a consequence and a marker of several cardiometabolic disorders such as obesity,^45–47^ insulin resistance, type 2 diabetes,^18,48–51^ while the glycine-to-valine ratio in amniotic fluid is associated with fetal growth restriction.^52^ A study conducted by White et al^45^ on the metabolic changes in Zucker-fatty rats on a tBCAA-restricted diet revealed increased concentrations of glycine in muscle tissue alongside decreased levels of acyl-CoA. These observations suggest that a scarcity of glycine, resulting from augmented tBCAA degradation, may lead to the harmful accumulation of acyl-CoA and impede the clearance of acyl groups through urinary excretion of acyl-glycine.^45,46^ Further investigations provided insights into the mechanisms driving the inverse relationship between glycine and tBCAA levels. Whether plasma tBCAAs increment causes glycine depletion, resulting in hypertension, remains to be established. Nonetheless, the above studies indicate that glycine metabolism should be considered in conjunction with tBCAA metabolism, which further justifies the analytical utility of this metabolite ratio as a potent risk factor for essential hypertension and associated cardiovascular pathologies such as CHD instead of a potentially less powerful assessment of the individual metabolites.

Additionally, the utilization of metabolite ratios rather than absolute concentrations presents significant analytical advantages, notably reducing variability within data sets.^26^

Further in vivo studies should address the molecular mechanisms of glycine and tBCAAs and their interplay in hypertension and CHD, beyond the abovementioned links with type 2 diabetes and insulin resistance. Possible mechanisms may revolve around the link between the levels of circulating tBCAAs and the development of inflammation and oxidative stress within endothelial cells, which in turn can lead to endothelial dysfunction.^22,53^ Additionally, elevated levels of circulating tBCAAs trigger the continuous activation of the mTOR pathway that may increase BP by initiating a range of alterations, including increased synthesis of glucosamine (an inhibitor of endothelial nitric oxide signaling),^54^ enhanced proliferation of vascular smooth muscle cells,^55^ changes in sympathetic nerve activity,^56^ and the risk of kidney damage.^8,57,58^ In contrast, beneficial effects of glycine might be driven by its anti-inflammatory and antioxidant properties, which possibly arise from its potential to inhibit NF-κB (nuclear factor kappa B) signaling in vascular endothelium and to inhibit production of proinflammatory cytokines by immune cells, as well as the fact that glycine serves as a substrate for glutathione synthesis, a major cellular antioxidant.^23,44,59^ Furthermore, endothelial cells express glycine-gated chloride channels that, upon glycine binding, prompt membrane hyperpolarization, which in turn facilitates the synthesis of nitric oxide, a potent vasodilator, inhibitor of platelet adhesion and aggregation, and anti-inflammatory agent, thereby offering a protective mechanism against atherosclerosis.^18,60^ While all the above mechanisms are in line with and might potentially explain increased tBCAAs/glycine ratio in hypertension/CHD conditions, they have to be examined in future animal studies using in vivo models.

The limitations of the current study may relate to the origin of the cohorts used and the interpretation of results from genetic causal inference tests. The cohorts involved in the current study were primarily of White ethnicity, which potentially limits the translation of the findings to other ethnic groups. While randomized controlled trials remain a state-of-the-art design type to address causal pathways leading to the development of various diseases, MR studies may provide additional evidence above purely observational results.^15^ Since assumptions of MR analysis are often untestable, we ensured that IVs were strongly associated with amino acid levels, and genetic estimates were adjusted for population structure. We have also used various sensitivity MR methods, allowing relaxing these assumptions, which demonstrated similar effect sizes and directions regarding most of the associations reported. Similar conclusions were derived from prospective analyses using a large cohort and confirmed the direction of the effect of amino acid traits on the development of hypertension and CHD.

### Perspectives

A combination of MR and observational prospective studies suggests a potentially causal link between glycine and tBCAAs and the risk of developing hypertension or CHD. The identified metabolic signature, marked by concurrently increased levels of tBCAAs and reduced glycine, could represent a valuable plasma clinical biomarker for identifying individuals at risk of hypertension and CHD. This signature may also indicate underlying biochemical pathways shared by both types of amino acids.

## ARTICLE INFORMATION

### Acknowledgments

This research has been conducted using the UK Biobank Resource under Application Number 93156.

### Sources of Funding

This study was funded in part by National Science Centre, Poland (grant number 2022/45/B/NZ4/00442 to M. Siedlinski), European Research Council (InflammaTENSION; ERC-CoG-726318; to T.J. Guzik), British Heart Foundation (FS/14/49/30838 and FS/4yPhD/F/20/34127A to T.J. Guzik), and European Commission and the National Center for Research and Development (Poland; ERA-CVD/JTC2020/25/ImmuneHyperCog/2022 and ERA-CVD/Gut-brain/8/2021 to T.J. Guzik). For the purpose of Open Access, the author has applied a CC-BY public copyright license to any Author Accepted Manuscript version arising from this submission.

### Disclosures

None.

### Supplemental Material

Figures S1–S7

Tables S1–S7

## Supplementary Material
