# Supplementary material for: Interplay Between Plasma Glycine and Branched-Chain Amino Acids Contributes to the Development of Hypertension and Coronary Heart Disease
Source: Hypertension. 2024 Apr 8;81(6):1320–31. doi: 10.1161/HYPERTENSIONAHA.123.22649 (PMC11095885; doi:10.1161/HYPERTENSIONAHA.123.22649)
Supplement: Supplementary file 1 [file hyp-81-1320-s001.pdf]

Online supplementary methods and figures

An interplay between plasma glycine and branched-chain amino acids contributes to the development of hypertension and coronary heart disease.

Mateusz Dziedzic, MSc[1], Ewelina Józefczuk, PhD[1], Tomasz J. Guzik, MD, PhD[1,2,3] and Mateusz Siedlinski, PhD[1,2,3]

[1]Department of Internal Medicine, Faculty of Medicine, Jagiellonian University Medical College, Cracow, Poland
[2]Center for Medical Genomics OMICRON, Faculty of Medicine, Jagiellonian University Medical College, Cracow, Poland
[3]Centre for Cardiovascular Science, Queen's Medical Research Institute, University of Edinburgh, Edinburgh, United Kingdom

Methods

Blood pressure measurement procedure in the UK Biobank study

The participant was collected from the waiting area and was seated in a curtained office. The participant took the first part of the interview questionnaire. After completing this, the staff member informed the participant that they would now take the first of two measurements of blood pressure. The participant was asked to sit with their feet parallel to each other, toes pointing forward and soles of feet flat on the floor. There should be no restrictive clothing to impede the circulation to their left upper arm: the participant was asked to loosen or remove any restrictive clothing. The right arm was only used if the left was not practical: e.g. amputee, shunt, mastectomy, axiliary clearance). Since resting blood pressure was measured, the staff member took care not to engage the participant in conversation. A Seca tape measure was used to determine the circumference of midpoint of upper arm, and the appropriate size of blood pressure cuff was selected. The appropriate sized cuff was put on the upper arm of participant (it might, depending on size of upper arm, encircle the arm several times). The cuff was rotated so that the green marker tab indicating the centre of the cuff bladder lied over the brachial artery, which was located at the inner part of front of the elbow. The rubber inflation tubing sit over the brachial artery in line with the participant's middle finger, and the bottom of cuff sit 1-2 cm above the elbow joint. When the cuff was correctly positioned it was secured with Velcro fasteners. The participant was asked to place their arm on the desk top, so that the cuff was at about same level as their heart, and to breathe in and out slowly five times in a relaxed fashion. The rubber inflation tubing was connected to the Omron blood pressure monitor and, „Start" on the monitor was pressed. When the blood pressure result was displayed on monitor, the „Retrieve" button on the computer was selected to transfer results from the monitor and then „Accept" to complete the first measurement. If there was a problem with the measurement, „Reject" was selected and the measurement repeated. If the largest cuff size was too small for the participant, or if the electronic blood pressure monitor failed to produce a reading, a sphygmomanometer with an inflatable cuff was used in conjunction with a stethoscope. The IT system prompted the staff member to scan the sphygmomanometer by using the barcode reader before taking the measurement. After completing the first measurement, the rubber inflation tubing was disconnected from the Omron monitor, with the cuff left in place. The participant was asked to gently shake their arm, and open and close their hand a few times. A timer in the computer ensured that the second blood pressure reading could not be taken until at least 1 minute had elapsed. This rest period was used to complete the second part of the interview. The inflation tubing was reconnected to the Omron blood pressure monitor and a second reading was made using the same procedure as before. Then the cuff was removed.

More details can be found at:
http://biobank.ndph.ox.ac.uk/showcase/showcase/docs/Bloodpressure.pdf

Genome-wide association studies in the UK Biobank

GWAS on SBP/DBP level was performed on 136,111 unrelated, Caucasian UK Biobank participants who were not included in the GWAS on amino acid levels with adjustment for age, sex, top 10 principal components for genetic ancestry and genotyping batch (UK Biobank vs UK BiLEVE). Subjects with missing genotype rate>10% were excluded. SBP/DBP values from 2 automated readings were averaged

and adjusted for subjects reporting anti-hypertensive medication use (UK Biobank field ids: 6177 and 6153) by adding 15/10 mmHg respectively.[1] GWAS on CHD (UK Biobank field IDs: 131296-131307; 19,563 cases and 131,305 controls) and HTN (UK Biobank filed IDs: 131286-131287; 59,735 cases and 89,720 controls with no BP-lowering treatment at baseline) were performed on unrelated, Caucasian UK Biobank participants who were not included in the GWAS on amino acid levels with adjustment for age, sex, top 10 principal components for genetic ancestry and genotyping batch (UK Biobank vs UK BiLEVE). In this way, ICD10-based definition of HTN and CHD, applied in UK Biobank GWAS, was similar to the one used in the FinnGen consortium[2], which was additionally used for (meta) analyses in the current study. No significant (nominal p value<0.05) differences between subjects included in the GWAS on amino acid level and remaining unrelated, Caucasian UK Biobank subjects were observed with respect to baseline age or SBP level (mean of 56.90±7.98 vs 56.86±8.00 years and 138.25±18.56 vs 138.14±18.60 mmHg respectively; analyzed with t-test) and sex (46.4% vs 46,2% males respectively, analysed using chi-square test), while marginal, yet nominally significant, differences were observed with respect to DBP level and BMI (mean of 82.18±10.07 vs. 82.33±10.09 mmHg and 27.43±4.74 vs. 27.36±4.76 kg/m^2 respectively).

REFERENCES

1. Evangelou E, Warren HR, Mosen-Ansorena D, Mifsud B, Pazoki R, et al. Genetic analysis of over 1 million people identifies 535 new loci associated with blood pressure traits. *Nat Genet*. 2018;50:1412–1425.

2. Kurki MI, Karjalainen J, Palta P, Sipilä TP, Kristiansson K, et al. FinnGen provides genetic insights from a well-phenotyped isolated population. *Nature*. 2023;613:508–518.

Figure S1: Kaplan-Meier analysis of incident HTN among normotensive subjects and CHD according to amino acid quintiles in the UK Biobank

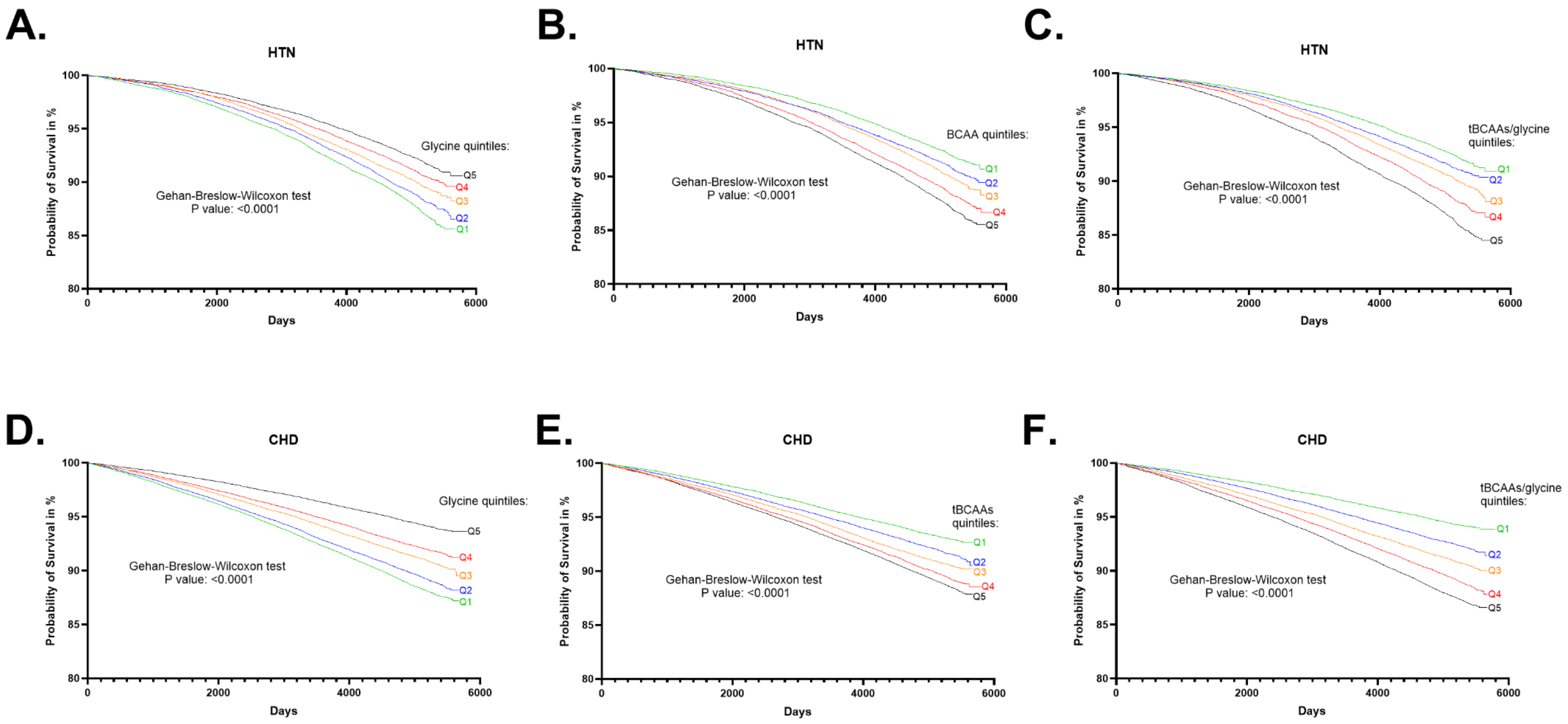

Survival plots were generated for HTN (A-C) and CHD (D-F) according to quintiles of glycine (A,D) tBCAAs (B,E) and tBCAAs/glycine ratio (C,F).

Figure S2: Risk to develop HTN among normotensive subjects and CHD according to amino acid quintiles in the UK Biobank adjusted for additional covariates

A.

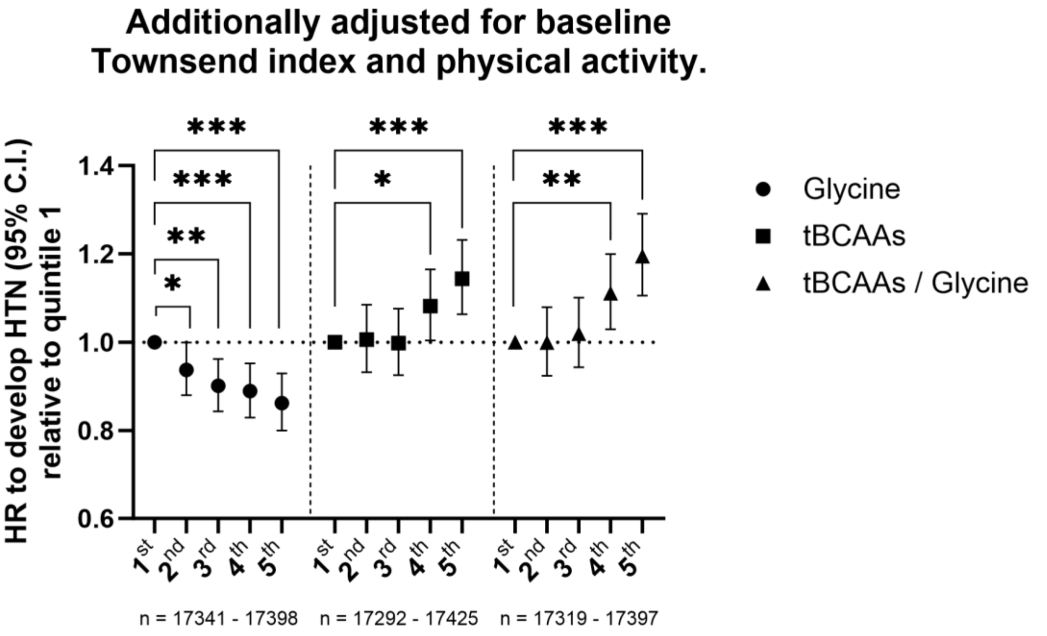

B.

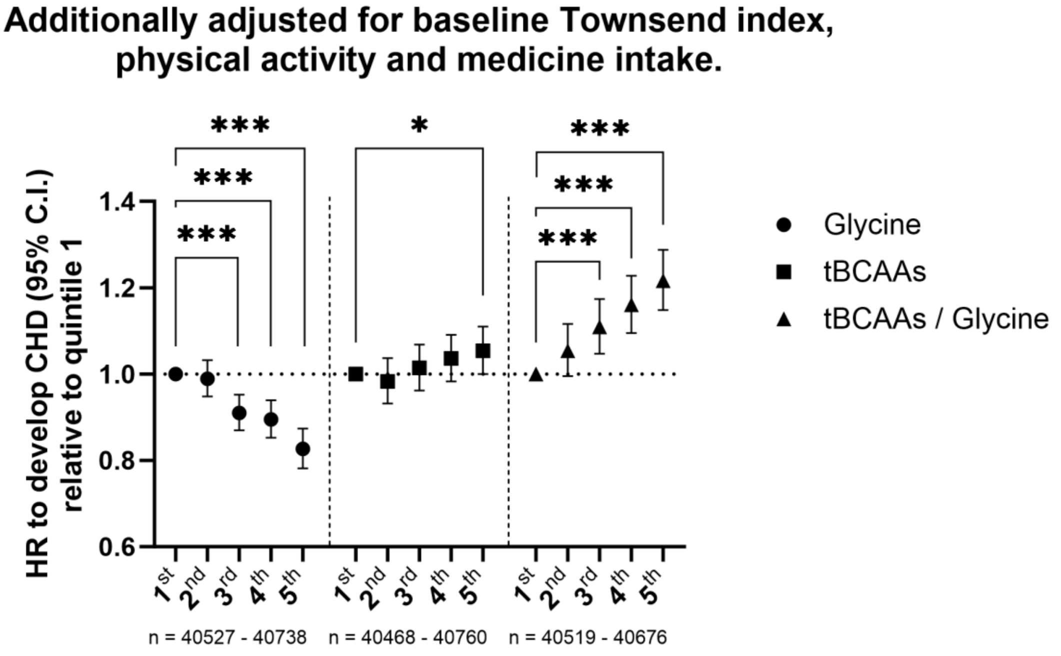

C.

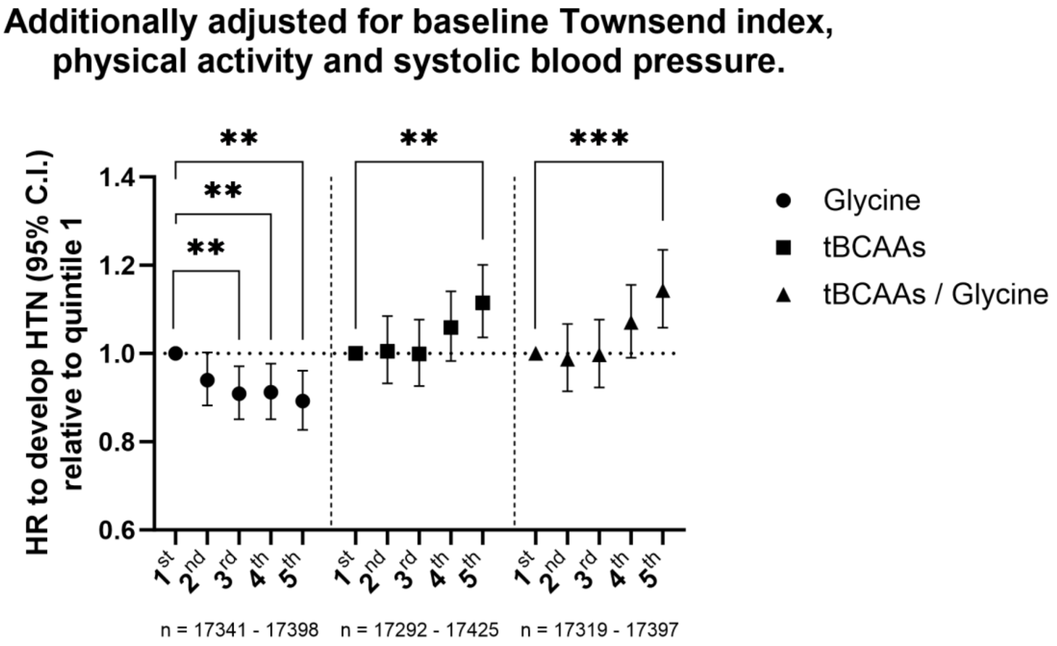
Additionally adjusted for baseline Townsend index, physical activity and systolic blood pressure.
HR to develop HTN (95% C.I.) relative to quintile 1
1.4
1.2
1.0
0.8
0.6
**
**
**
**
***
1st 2nd 3rd 4th 5th
n = 17341 - 17398
n = 17292 - 17425
n = 17319 - 17397
Glycine
tBCAAs
tBCAAs / Glycine

D.

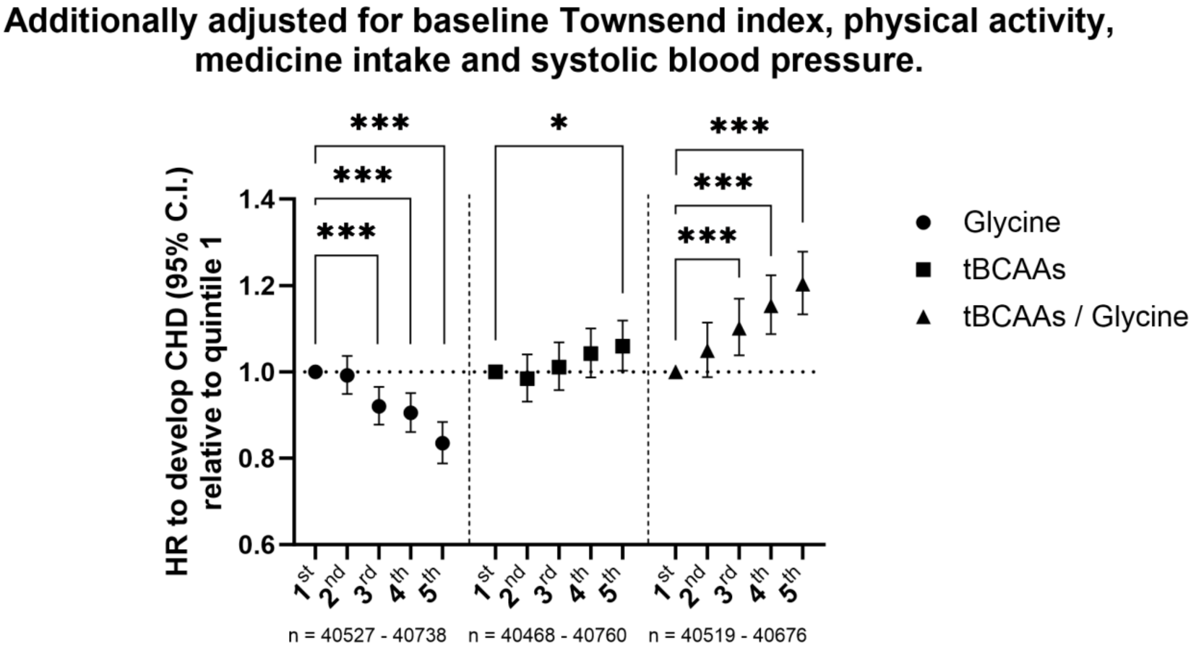
Additionally adjusted for baseline Townsend index, physical activity, medicine intake and systolic blood pressure.
HR to develop CHD (95% C.I.) relative to quintile 1
1.4
1.2
1.0
0.8
0.6
***
***
***
*
***
***
***
1st 2nd 3rd 4th 5th
n = 40527 - 40738
n = 40468 - 40760
n = 40519 - 40676
Glycine
tBCAAs
tBCAAs / Glycine

A. Analyses were performed in 89,845 normotensive participants (average automated SBP and DBP readings below 140 and 90mmHg respectively, no BP-lowering treatment at baseline) using Cox regression adjusted for sex, age, BMI, smoking status, alcohol consumption, physical activity, and Townsend deprivation index.

   HR = Hazard ratio
   C.I. = Confidence Interval
   tBCAAs = total branched-chain amino acids
   HTN = essential hypertension
   *p < 0.05, ***p < 0.001

B. Analyses were performed in all UK Biobank participants with data on amino acid level available using Cox regression adjusted for sex, age, BMI, smoking status, alcohol consumption, physical activity, Townsend deprivation index and intake of medications (insulin, BP-lowering and cholesterol-lowering).

   HR = Hazard ratio
   C.I. = Confidence Interval
   tBCAAs = total branched-chain amino acids
   CHD = coronary heart disease
   *p < 0.05, ***p < 0.001

C. Analyses were performed in 89,845 normotensive participants (average automated SBP and DBP readings below 140 and 90mmHg respectively, no BP-lowering treatment at baseline) using Cox regression adjusted for sex, age, BMI, smoking status, alcohol consumption, physical activity, Townsend deprivation index, and systolic blood pressure.

   HR = Hazard ratio
   C.I. = Confidence Interval
   tBCAAs = total branched-chain amino acids
   HTN = essential hypertension
   **p < 0.01, ***p < 0.001

D. Analyses were performed in all UK Biobank participants with data on amino acid level available using Cox regression adjusted for sex, age, BMI, smoking status, alcohol consumption, physical activity, Townsend deprivation index, intake of medications (insulin, BP-lowering and cholesterol-lowering) and systolic blood pressure.

   HR = Hazard ratio
   C.I. = Confidence Interval
   tBCAAs = total branched-chain amino acids
   CHD = coronary heart disease
   *p < 0.05, ***p < 0.001

Figure S3: Hazard ratio of developing essential HTN and CHD according to quintiles of both tBCAAs and Gly

A.

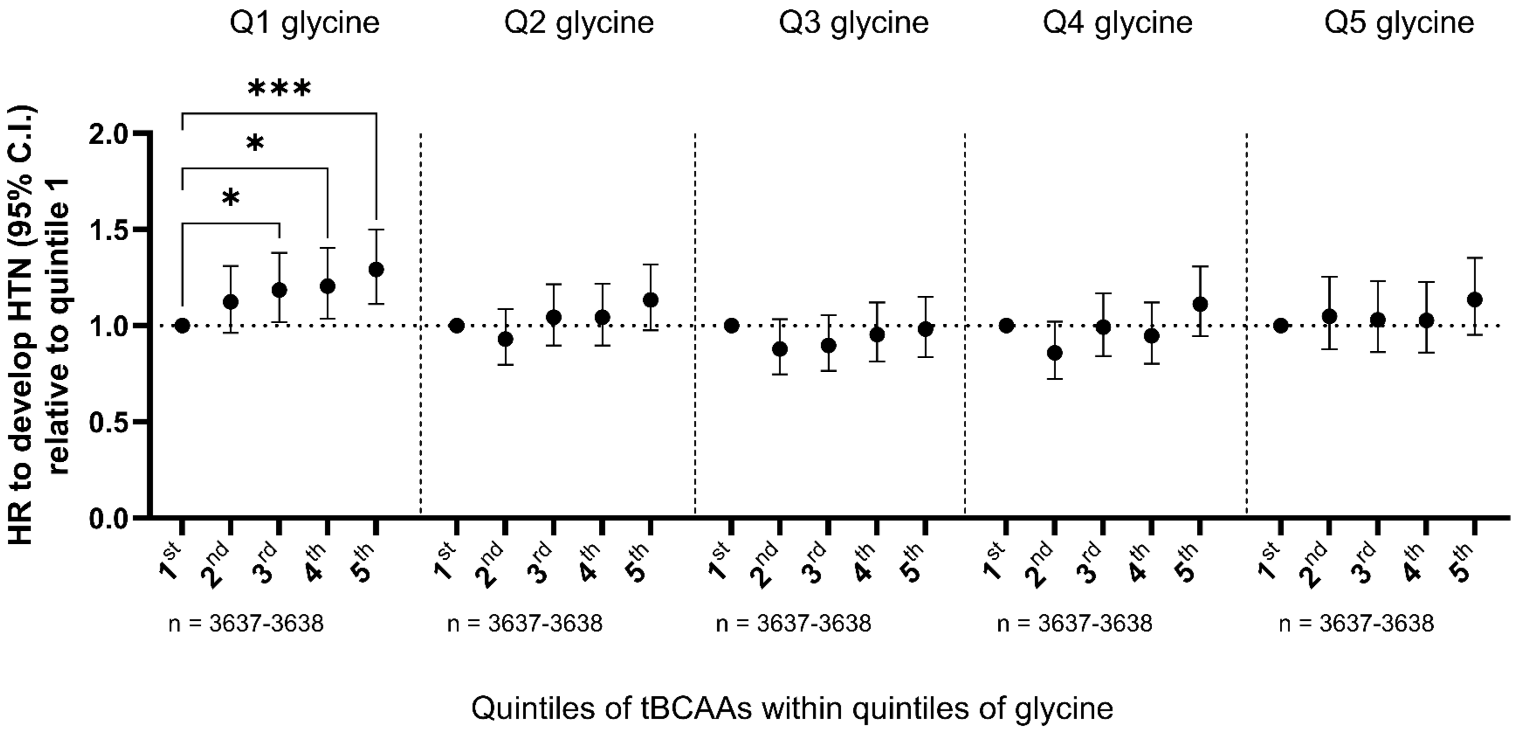

B.

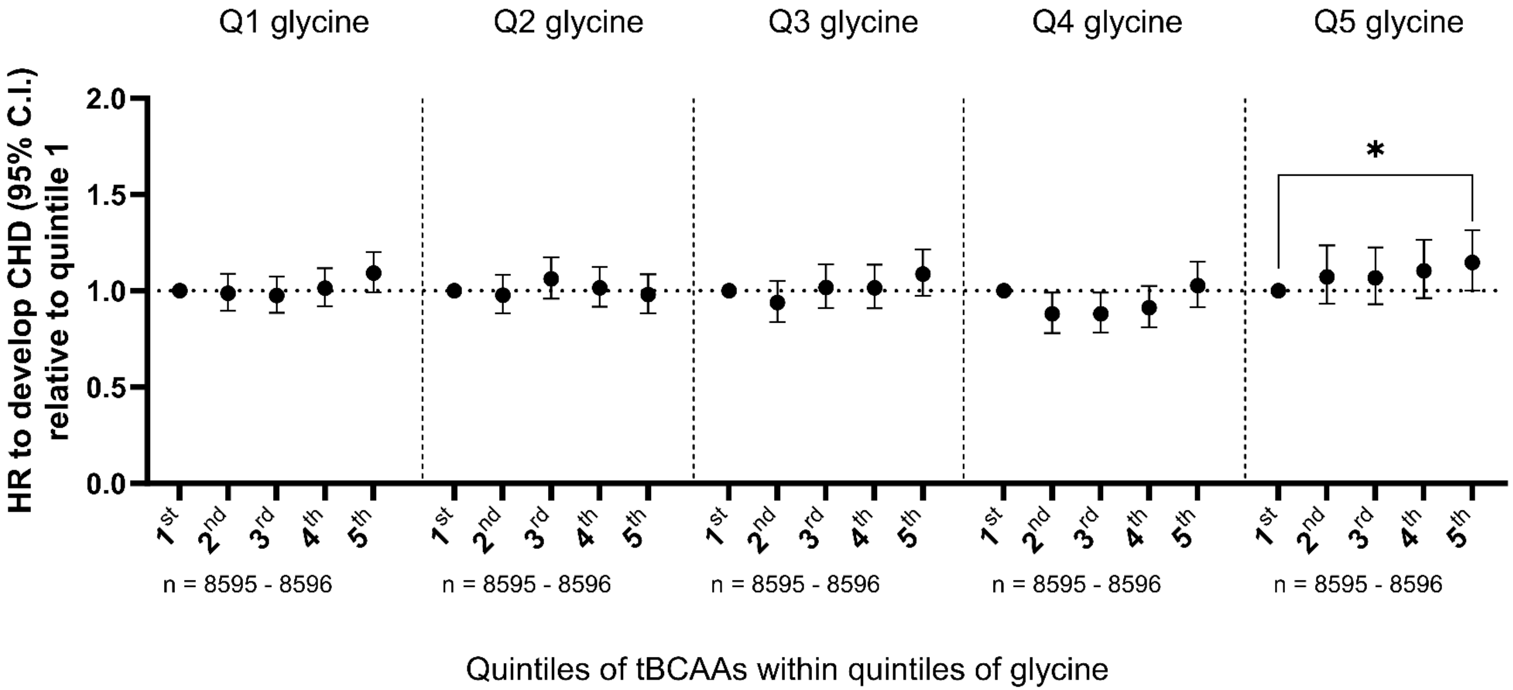

Q1 glycine
Q2 glycine
Q3 glycine
Q4 glycine
Q5 glycine
HR to develop CHD (95% C.I.) relative to quintile 1
2.0
1.5
1.0
0.5
0.0
*
1st 2nd 3rd 4th 5th
n = 8595 - 8596
Quintiles of tBCAAs within quintiles of glycine

A. Analyses were performed in normotensive participants (average automated SBP and DBP readings below 140 and 90mmHg respectively, no BP-lowering treatment at baseline) using Cox regression adjusted for sex, age, BMI, smoking status and alcohol intake frequency.
   HR = Hazard ratio
   C.I. = Confidence Interval
   tBCAAs = total Branched-chain amino acids
   Gly = glycine
   HTN = essential hypertension
   Q1-5 = quantiles of glycine
   *p < 0.05, ***p < 0.001

B. Analyses were performed in all UK Biobank participants with data on amino acid level available using Cox regression adjusted for sex, age, BMI, smoking status and alcohol intake frequency.,

   HR = Hazard ratio
   C.I. = Confidence Interval
   tBCAAs = total branched-chain amino acids
   Gly = glycine
   CHD = coronary heart disease
   Q1-5 = quantiles of glycine
   *p < 0.05

Figure S4: Risk to develop HTN among normotensive subjects in the UK Biobank according to sex after adjustment for various sets of covariates

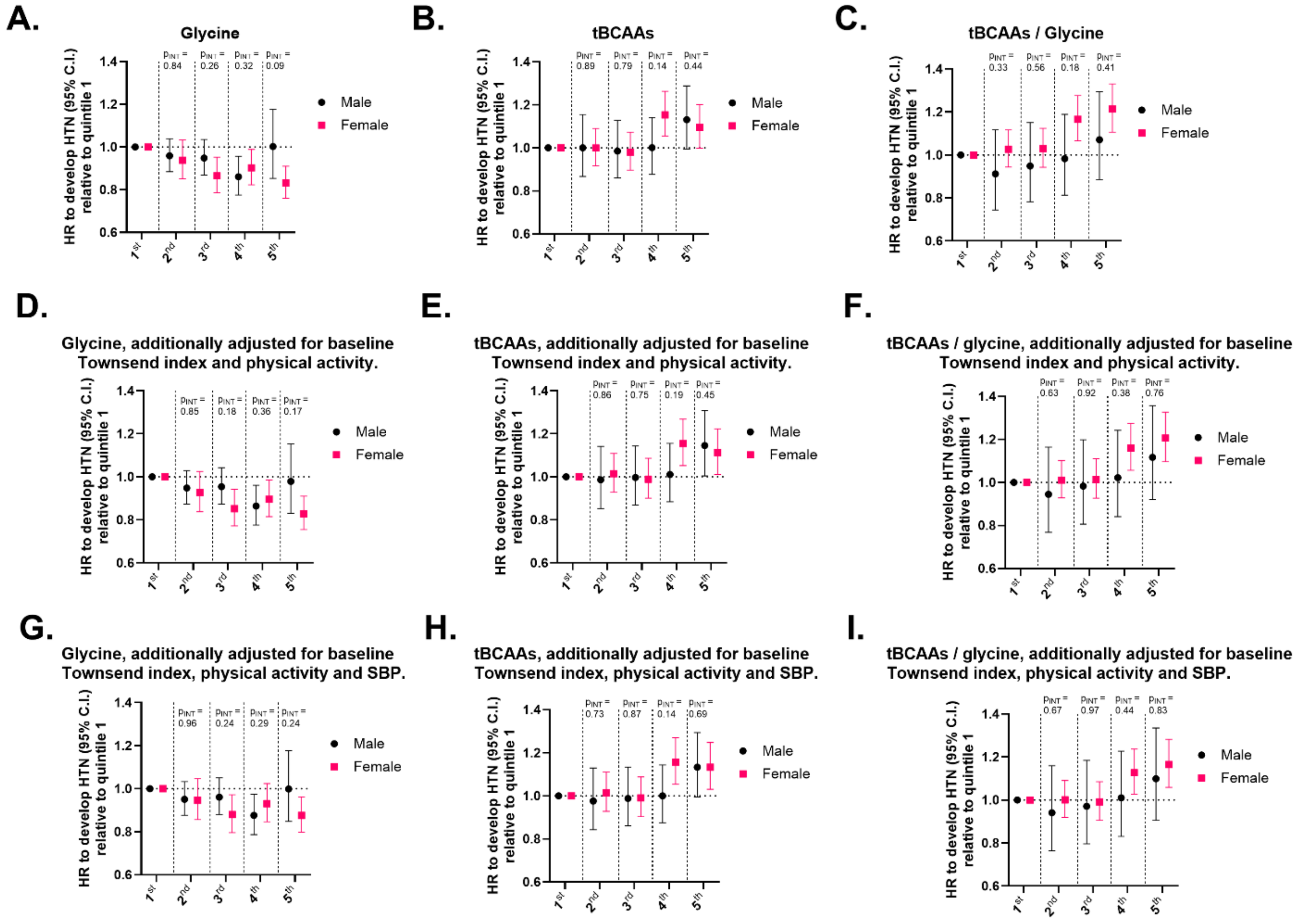

Depicted p value tested the effect of interaction between particular quintile and sex. Basic models (A, B, C) were adjusted for baseline age, BMI, smoking status and alcohol intake frequency.

Figure S5: Risk to develop CHD in the UK Biobank according to sex after adjustment for various sets of covariates

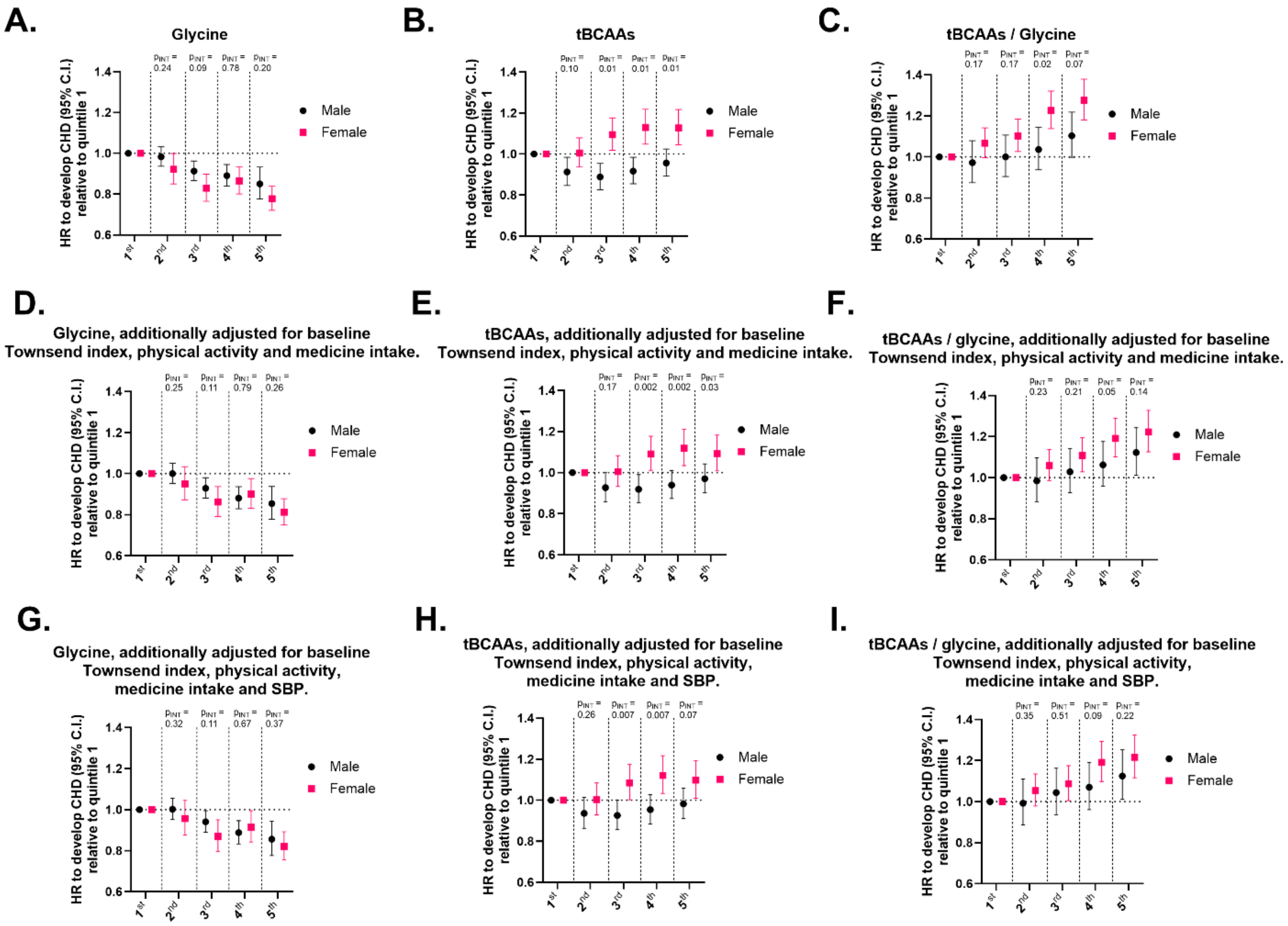

Depicted p value tested the effect of interaction between particular quintile and sex. Basic models (A, B, C) were adjusted for baseline age, BMI, smoking status and alcohol intake frequency.

Figure S6: Effects of genetically proxied amino acid traits on the level of BP and development of HTN – MR results using MR-PRESSO and MR-Egger approaches

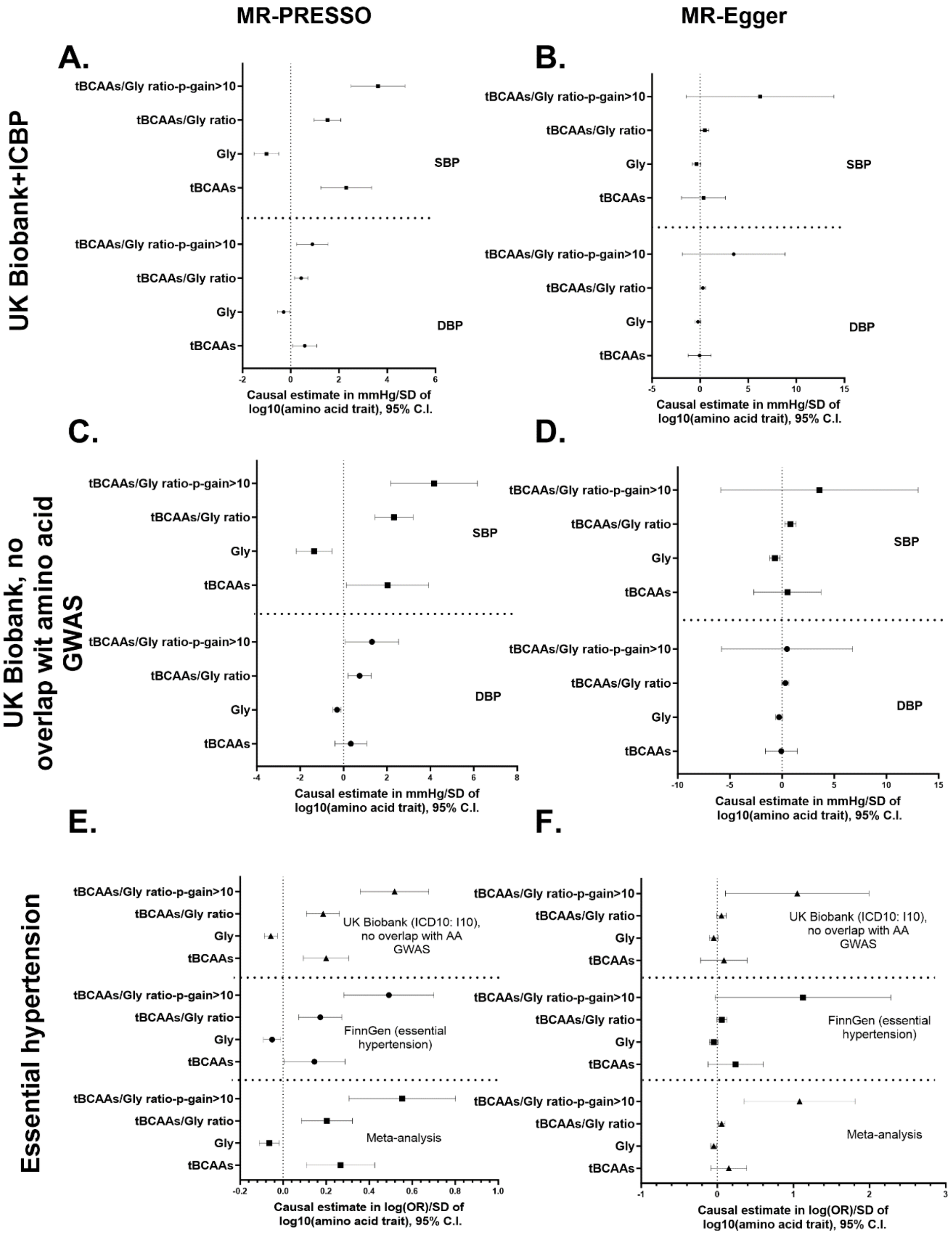

MR-PRESSO (A,C,E) and MR-Egger (B,D,F) Mendelian randomization methods were used to calculate causal estimates corresponding to the effects of Gly, tBCAAs and tBCAAs/Gly ratio on the level blood pressure and development of HTN. Analyses used independent (r^2<0.001) genetic instruments derived from GWAS performed in 186,523 unrelated, Caucasian UK Biobank participant (Supplementary Tables 2-5). A p-gain statistic>10 was used to identify genetic instruments specific for tBCAAs/Gly ratio. Outcome data were derived from meta-analysis of ICBP and UK Biobank (GWAS on SBP/DBP; A,B), UK Biobank (SBP/DBP (no overlapping subjects with amino acid GWAS); C,D) as well as UK Biobank (no overlapping subjects with amino acid GWAS) and FinnGen (HTN; E,F).

C.I. = Confidence Interval
tBCAAs = total branched-chain amino acids
HTN = essential hypertension
Gly=glycine
OR=odds ratio
SBP/DBP=systolic/diastolic blood pressure

Figure S7: Effects of genetically proxied amino acid traits on the development of CHD – MR results using MR-PRESSO and MR-Egger approaches

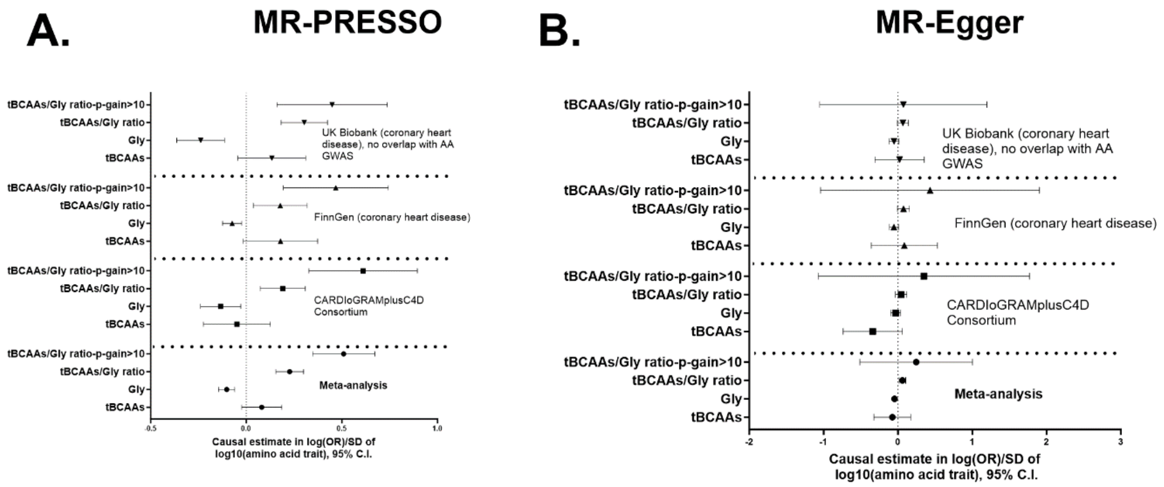

MR-PRESSO (A) and MR-Egger (B) Mendelian randomization methods were used to calculate causal estimates corresponding to the effects of Gly, tBCAAs and tBCAAs/Gly ratio on the development of CHD. Analyses used independent (r^2<0.001) genetic instruments derived from GWAS performed in 186,523 unrelated, Caucasian UK Biobank participant (Supplementary Tables 2-5). A p-gain statistic>10 was used to identify genetic instruments specific for tBCAAs/Gly ratio. Outcome data were derived from UK Biobank (no overlapping subjects with amino acid GWAS) FinnGen and CARDIoGRAMplusC4D consortium.

C.I. = Confidence Interval
tBCAAs = total branched-chain amino acids
CHD = coronary heart disease
Gly=glycine
OR=odds ratio
